# Transient Ischemic Attacks Preceding Ischemic Stroke and the Possible Preconditioning of the Human Brain: A Systematic Review and Meta-Analysis

**DOI:** 10.3389/fneur.2021.755167

**Published:** 2021-11-24

**Authors:** Sherief Ghozy, Salah Eddine Oussama Kacimi, Mohamed Elfil, Mohamed Gomaa Sobeeh, Abdullah Reda, Kevin M. Kallmes, Alejandro A. Rabinstein, David R. Holmes, Waleed Brinjikji, Ramanathan Kadirvel, David F. Kallmes

**Affiliations:** ^1^Department of Radiology, Mayo Clinic, Rochester, MN, United States; ^2^Faculty of Medicine, University of Tlemcen, Tlemcen, Algeria; ^3^Department of Neurological Sciences, University of Nebraska Medical Center, Omaha, NE, United States; ^4^Faculty of Physical Therapy, Cairo University, Cairo, Egypt; ^5^Faculty of Physical Therapy, Sinai University, Cairo, Egypt; ^6^Faculty of Medicine, Al-Azhar University, Cairo, Egypt; ^7^Nested Knowledge, St. Paul, MN, United States; ^8^Superior Medical Experts, St. Paul, MN, United States; ^9^Department of Neurology and Neurocritical Care, Mayo Clinic, Rochester, MN, United States; ^10^Department of Cardiovascular Diseases and Internal Medicine, Mayo Clinic, Rochester, MN, United States; ^11^Department of Neurosurgery, Mayo Clinic Rochester, Rochester, MN, United States

**Keywords:** ischemic attack, transient, stroke, neuroprotection, ischemic preconditioning, meta-analysis

## Abstract

Stroke is a leading cause of mortality and disability worldwide. Transient ischemic attack (TIA) is defined as transient brain ischemia with temporary neurological deficits. In animal models, prior TIA seems to enhance brain ischemic tolerance to withstand further ischemic events, which might be explained by brain preconditioning. Thus, this review aims to formulate evidence of whether TIAs can induce positive preconditioning and enhance the functional outcomes in patients suffering from subsequent ischemic strokes. Five databases were searched (PubMed, Embase, SAGE, Web of Science, and Scopus), and twelve studies were included in the quantitative analysis. Studies were eligible when comparing patients with acute ischemic stroke (AIS) and previous TIA with those with AIS without TIA. Comparisons included the National Institute of Health Stroke Scale (NIHSS) score at admission and 7 days from the stroke event, modified Rankin score (mRS), and Trial of ORG 10,172 in Acute Stroke Treatment (TOAST) classification. Odds ratio (OR), mean difference (MD), and 95% confidence interval (CI) were used to describe our results using the random effect model. Our results revealed that patients with stroke and prior TIAs had lower NIHSS scores at admission than those without prior TIAs. However, the NIHSS score was not significantly different between the two groups at 7 days. Furthermore, there was no statistically significant difference between both groups in terms of mortality. Despite the differences in the admission mRS score groups, patients with prior TIAs had lower mRS scores at discharge.

## Introduction

Stroke is one of the most common causes responsible for mortality, morbidity, and loss of function worldwide (1). Furthermore, it has been reported that after the age of 55, the risk of having stroke doubles per each subsequent decade, with overburdened healthcare economics for these patients (2). Consequently, many management approaches have been extensively reported in the literature, which mainly target decreasing the incidence of severe events and mortality after a stroke event, in addition to enhancing recovery and rehabilitation among the survivors (3–6).

Although ischemia accounts for a significant part of brain tissue injury during stroke, reperfusion also contributes to brain tissue damage (7). Many explanations have been proposed to explain the underlying pathophysiology of the reperfusion injury, including the potential dysregulation of the intracellular calcium (8), impaired ion transport across the plasma membrane (8, 9), and increased synthesis and release of pro-inflammatory cytokines and reactive oxygen species (10, 11), all of which can cumulatively lead to the opening of the mitochondrial permeability transition pores resulting into tissue necrosis (12, 13). Thus, many approaches have been suggested to regulate the process of ischemia-reperfusion and enhance the clinical and prognostic outcomes. Ischemic preconditioning (IPC) has shown a potential as a modality in reducing the severity of the post-ischemic events. However, controversies still exist in literature about the potential neuroprotective effects that this approach might have (14).

Ischemic tolerance has been primarily described in experimental studies as a process during which the brain is subjected to a short period of ischemia which might enable the brain to be more tolerant when exposed to a more persistent ischemic event (15–19). Therefore, it has been proposed that pre-stroke exposure to Transient ischemic attacks (TIAs) might have a potential neuroprotective role when persistent ischemia affects the same exposed region. However, clinical studies reported contradicting findings regarding the ability of TIAs to alleviate the severity of cerebral ischemic injury (20–23). Accordingly, we aimed to conduct this systematic review and meta-analysis to formulate evidence whether TIAs can induce positive brain preconditioning and enhance the functional outcomes in patients suffering from subsequent AIS.

## Methods

### Search Strategy and Study Selection

We conducted this study following the recommendations of the Preferred Reporting Items of Systematic Reviews and Meta-analysis (PRISMA) checklist (24). We searched for eligible papers till 1^st^ June 2021 in five databases listed in order: PubMed, Web of Science (ISI), Scopus, SAGE, and Embase databases using keywords, medical subject (MeSH) terms, and publication types based on the patient/population, intervention, comparison, and outcomes (PICO) framework. Participants were any patient with prior history of TIAs who developed AIS later, and there was no specific intervention applied rather than the ordinary care. The comparison group included patients who developed AIS with no known history of prior TIAs, and all reported outcomes were analyzed whenever possible.

Although we tailored the search strategy based on each database, the following search terms were the main components: (“Ischemic attack” OR “transient ischemic attack” OR “TIA^*^” OR “brain TIA” OR “posterior circulation transient ischemic attack” OR “anterior circulation transient ischemic attack” OR “transient cerebral ischemia” OR “transient brainstem ischemia”) AND (“stroke” OR “CVA” OR “CVS” OR “cerebrovascular accident^*^” OR “cerebrovascular stroke” OR “hemorrhagic stroke” OR “thrombotic stroke” OR “cerebral infarction” OR “acute stroke^*^” OR “cerebral stroke^*^” OR “brainstem infarction” OR “lacunar infarction” OR “ischemic stroke” OR “lacunar stroke” OR “cardio-embolic stroke” OR “brainstem ischemia”) AND (“Ischemic preconditioning” OR “ischemic preconditioning” OR “ischemic preconditioning” OR “preconditioning”). In addition, we did a manual search of references in the included papers to avoid missing relevant studies (25). Finally, we included all original studies with no publication date or language restrictions to prevent bias from missing any relevant papers.

We excluded records or full texts that included animal populations, non-original studies, case reports, and case series. We also excluded studies that reported on the same patients because they will lead to results bias. Three authors screened the title and abstracts of each record then performed full-text screening to include relevant papers. In each step, a fourth reviewer revised the screening results to ensure more accuracy. Finally, a senior author was consulted if disagreement occurred.

### Data Extraction

We extracted the data using a pre-designed Excel sheet. At least two investigators extracted the necessary information from each paper and revised it by a third investigator for more accuracy.

### Quality Assessment for the Included Studies

Two authors independently used the Newcastle-Ottawa Scale (NOS) to evaluate the quality of the included studies (26). The scale consists of the quality of selection (representativeness of the exposed cohort, selection of the non-exposed cohort, ascertainment of exposure, and demonstration that the outcome measure was not present at the start of study); comparability (comparability of cohorts based on the design or analysis); and outcome (assessment of the outcome, if the follow-up period was long enough for outcomes to occur, and adequacy of the follow-up of cohorts) (27). If there were any disagreements, it was resolved by a third author.

### Statistical Analysis

All data were analyzed using R software version 4.0.2 (28). Using the “meta” package, we computed the pooled odds ratios (ORs) or mean differences (MDs) and their corresponding 95% confidence intervals (CIs), using a random-effects model due to the heterogenous methodologies among the included studies. Heterogeneity was assessed with Q statistics and *I*^2^-test considering it significant with *I*^2^-value >50% or *P*-value <0.05 (29, 30). Due to the small number of the included studies (<10 per the analysis), neither Egger's regression test for the assessment of publication bias nor meta-regression were possible (31, 32).

## Results

### Search Results

**Figure 1:**
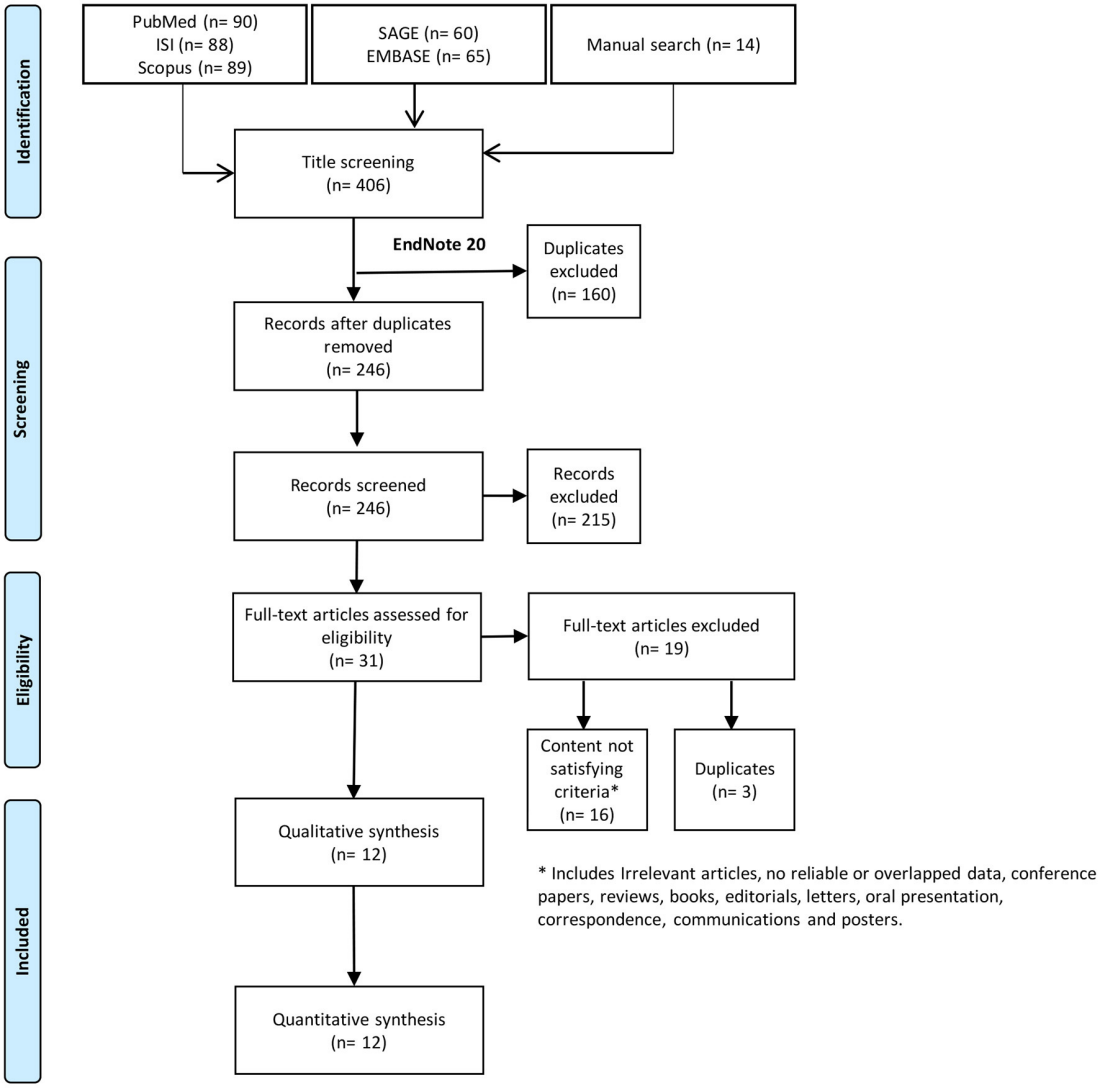
Study flow diagram.

Systematic search resulted in 406 records; 246 of these records were screened using title and abstract after excluding duplicates. Title and abstract screening yielded 31 papers eligible for full-text screening. Out of these, we included 12 studies in the current systematic review (Figure 1).

### Characteristics of the Included Studies and Patients

The retrospective study design was the most prevalent among eight of the included studies, where the sample sizes varied widely from only 65 to 7,611 patients. Four studies were conducted in Germany, four in Spain, two in Switzerland, one in Italy, and one in Hungary. Six studies used both mRS and NIHSS to assess the severity of the disability, seven studies reported the odds of cardioembolism and large vessel stroke in both groups, five studies reported the mortality rate, and three studies reported the odds of small vessel stroke. The characteristics of the included studies are summarized in Table 1. The mean age of all included patients was above 50 years, ranging from 56 to 75.7 years. Different special habits and co-morbidities were comparable among patients with prior TIAs and those without them (Table 2).

**Table 1:** Characteristics of the included studies.

**Study**	**Country (Study periode)**	**Study design**	**Sample size, (TIA/no TIA)**	**Exclusion criteria: participants with**	**Primary end point**	**Severity Measurement**	**Definition of favorable outcomes**
Arboix et al. (33)	Spain (1986–1997)	Retrospective	1,753 (221/1,532)	NR	Complications, mortality early recovery, length of stay	mRS	mRS 0–2
Weber et al. (34)	Germany (2002–2006)	Prospective	7,611 (7,159/452)	Previous stroke, aphasia, unconscious	Sevirity, mRS, NIH-SS at admission and discharge	NIH-SS, mRS	NA
Wegener et al. (23)	Germany (1997–2001)	Retrospective	65 (16/49)	ICH, older lesions on T2-weighted images insufficient data	MRI finding, mRS, NIH-SS at admission and discharge	NIH-SS, mRS	NA
Schaller et al. (35)	Switzerland (2000–2002)	Prospective	130 (11/119)	TIA >60 min	NIH-SS at admission and at follow-up, TIMI	NIH-SS	NA
Zsuga et al. (36)	Hungary (1996–2000)	Retrospective	2,201 (195/2,006)	NR	In-hospital mortality	Mortality	NA
Castillo et al. (37)	Spain (1999–2001)	Retrospective	283 (38/245)	lacunar infarction, lack of blood samples, intravenous t-PA, inclusion in neuroprotective clinical trials, absence of follow-up	Blood markers, Canadian Stroke Scale Infarct volume	Barthel index	Survival or Barthel index score >85
Moncayo et al. (22)	Switzerland (1979–1997)	Retrospective	2,379 (293/2,086)	TIA >60 min	Clinical findings at admission	Functional state levels 1 and 2	Functional state levels 1 and 2
Sitzer er al. (38)	Germany (1998–2000)	Retrospective	4,797 (332/4,465)	Stupor or coma	mRS, Barthel index	mRS, Barthel index	mRS <1 + Barthel index >90
Weih et al. (21)	Germany (1994–1998)	Retrospective	148 (37/111)	Hemorrhagic stroke, SAH, cerebral sinus thrombosis, CSS < 4 OR > 10.5 aphasia, dementia, previous stroke, previous TIA with infarction	Mortality, complications improvement	mRS	Glasgow coma scale score = 5
Della Morte et al. (39)	Italy (2000–2005)	Retrospective	203 (42/161)	Hemorrhagic stroke, SAH, cerebral sinus thrombosis, dementia, previous stroke, previous TIA with infarction	NIH-SS at admission and discharge	NIH-SS	NA
Alonso de Lecinana et al. (40)	Spain (2003–2009)	Prospective	877 (60/817)	Episodes not fulfilling definition of TIA (transient focal neurological deficit lasting <24 h) were not considered in analysis	NIH-SS at admission, 2 h, 24 h, and 7 days	NIH-SS	>8 points from baseline NIHSS
Colas-Campas et al. (41)	Spain (2011–2013, 2014–2016)	Prospective	477 (39/438)	Symptoms <24 h without AIS in the neuroimaging. (mRS) >3, duration of stroke symptoms more than 24 h and/or patients under 18 years old	NIH-SS at admission, 24 h, 7 days mRS at admission, 7 days, 90 days clinical findings, lab findings	NIH-SS, mRS	NA

*NR, not reported; NA, not available; NIHSS, The National Institutes of Health Stroke Scale; mRS, The Modified Rankin Scale; TIA, transient ischemic attack; tPA, tissue plasminogen activator; SAH, subarachnoid hemorrhage; ICH: intracerebral hemorrhage*.

**Table 2:** Baseline characteristics of the included patients.

**Source**	**Age, Mean** **±** **SD**		**Gender (males), %**		**Smoking habits, %**		**Alcoholism, %**			
	**TIA**	**No-TIA**	**TIA**	**No-TIA**	**TIA**	**No-TIA**	**TIA**	**No-TIA**		
Arboix et al. (33)	75.7 ± 10.1	74.8 ± 10.7	50.6	50.4	8.1	10.6	0.9	2.8		
Colas-Campas et al. (41)	67.82 ± 12.5 (TIA ≤24 h) 71.4 ± 11.3 (TIA 24 h−7 days)	74.7 ± 11.4	63.6 (TIA ≤24 h) 67.5 (TIA 24 h−7 days)	57.8	36.36 (TIA ≤24 h) 29,41 (TIA 24 h−7 days)	18.3	9.1 (TIA ≤24 h) 0.0 (TIA 24 h−7 days)	7.3		
Della Morte et al. (39)	75.43 ± 2.27	74.13 ± 0.87	45	56	9	62	14	63		
Alonso de Lecinana et al. (40)	63 (51; 74)a	71 (60; 77)a	70	53	36.7	24.8	NA	NA		
Moncayo et al. (22)	65.8 ± 13.2 (<10 min) 62.7 ± 12.6 (10–20 min) 63.5 ± 13.4 (21–60 min)	63.1 ± 15.3	67 (<10 min) 68 (10–20 min) 55 (21–60 min)	60	33 (<10 min) 42 (10–20 min) 41 (21–60 min)	32	NA	NA		
Schaller et al. (35)	59.7	61.5	45	48	18	16	NA	NA		
Sitzer et al. (38)	69.2 ± 12.1	69.5 ± 12.7	61.6	53.5	24.9	15.4	NA	NA		
Weber et al. (34)	66.1 ± 12.7	68.0 ± 13.4	62.8	57	25.5	22.6	NA	NA		
Wegener et al. (23)	56.0 (12.8)b	62.0 (22.5)b	62.5	71.4	68.8	30.6	NA	NA		
Weih et al. (21)	59.4 ± 13.3	60.0 ± 12.8	49	49	35	32	NA	NA		
Zsuga et al. (36)	68.02 ± 12.43	67.70 ± 13.06	55.9	51.3	25.47	32.49	NA	NA		
	**Hypertension**		**Diabetes**		**Hyperlipidemia**		**IHD**		**AF**	
	**TIA**	**No-TIA**	**TIA**	**No-TIA**	**TIA**	**No-TIA**	**TIA**	**No-TIA**	**TIA**	**No-TIA**
Arboix et al. (33)	56.5	54.6	23.5	22.1	19	18.3	11.8	15.6	28.5	31
Colas-Campas et al. (41)	63.6 (TIA ≤24 h) 64.7 (TIA 24 h−7 days)	75.8	22.73 (TIA ≤24 h) 23.5 (TIA 24 h−7 days)	27.4	54.6 (TIA ≤24 h) 29.4 (TIA 24 h−7 days)	43.8	9.1 (TIA ≤24 h) 0.0 (TIA 24 h−7 days)	11.6	22.7 (TIA ≤24 h) 5.9 (TIA 24 h−7 days)	25.3
Della Morte et al. (39)	76	66	32	30	47	53	43	32	12	14
Alonso de Lecinana et al. (40)	51.7	60.4	10	18.3	42.9	31.7	NA	NA	8.3	18.3
Moncayo et al. (22)	56 (<10 min) 46 (10–20 min) 47 (21–60 min)	48	12 (<10 min) 16 (10–20 min) 13 (21–60 min)	14	35 (<10 min) 30 (10–20 min) 24 (21–60 min)	22	24 (<10 min) 23 (10–20 min) 22 (21–60 min)	22	5 (<10 min) 5 (10–20 min) 11 (21–60 min)	12
Schaller et al. (35)	64	48	NA	9	9	18	45	35	18	19
Sitzer et al. (38)	69.3	71.3	28.4	28.7	NA	NA	NA	NA	NA	NA
Weber et al. (34)	75.4	74	23.2	26.5	40.5	31.4	19.5	19.4	14.4	16.1
Wegener et al. (23)	43.8	63.3	12.5	32.7	50	36.7	NA	NA	NA	NA
Weih et al. (21)	62	60	24	25	35	28	14	18	16	18
Zsuga et al. (36)	67.69	56.13	16.41	15.85	NA	NA	NA	NA	18.56	11.43
**Source**	**PVD**		**Previous stroke**		**Anticoagulant**		**Contraception**		**COPD**	
	**TIA**	**No-TIA**	**TIA**	**No-TIA**	**TIA**	**No-TIA**	**TIA**	**No-TIA**	**TIA**	**No-TIA**
Arboix et al. (33)	11.8	7.4	13.1	17.2	NA	NA	NA	NA	NA	NA
Colas-Campas et al. (41)	NA	NA	NA	NA	13.6 (TIA ≤24 h) 5.9 (TIA 24 h−7 days)	12.8	NA	NA	NA	NA
Della Morte et al. (39)	29	24	NA	NA	55	60	NA	NA	19	28
Alonso de Lecinana et al. (40)	NA	NA	8.3	8.9	10	2.9			NA	NA
Moncayo et al. (22)	5 (<10 min) 6 (10–20 min) 4 (21–60 min)	7	NA	NA	NA	NA	0 (<10 min) 1 (10–20 min) 1 (21–60 min)	3	NA	NA
Schaller et al. (35)	9	1	NA	NA	NA	NA	9	4	NA	NA
Sitzer et al. (38)	NA	NA	NA	NA	NA	NA	NA	NA	NA	NA
Weber et al. (34)	NA	NA	NA	NA	NA	NA	NA	NA	NA	NA
Wegener et al. (23)	6.3	10.2	NA	NA	18.8	24.5	NA	NA	NA	NA
Weih et al. (21)	11	7	NA	NA	14	12	NA	NA	NA	NA
Zsuga et al. (36)	11.79	14.21	NA	NA	NA	NA	NA	NA	NA	NA

*NA, not available*.

### Quality Assessment for the Included Studies

The risk of bias assessment results was represented in Table 3. The agreement between the two reviewers was 65.4%, which is “substantial” (42). The minimum quality score of the included articles was six, while the maximum quality score was eight out of nine points (the maximum possible score). Thus, all of the included studies were of good quality. The third independent researcher was not required for making the final decisions.

**Table 3:** Quality assessment of the included studies.

**Author**	**Selection**				**Comparability**	**Outcome**			**Total score**
	**Representativeness of the exposed cohort**	**Selection of the non-exposed cohort**	**Ascertainment of exposure**	**Demonstration that outcome of interest was not present at start of study**	**Control for important or additional factors**	**Assessment of outcome**	**Was follow up long enough for outcomes to occur**	**Adequacy of follow up of cohorts**	
Arboix et al. (33)	⋆	⋆	⋆	⋆	⋆	⋆	⋆	⋆	8
Colas-Campas et al. (41)		⋆	⋆	⋆	⋆⋆	⋆	⋆	⋆	8
Castillo et al. (37)		⋆	⋆	⋆	⋆	⋆	⋆	⋆	7
Della Morte et al. (39)		⋆	⋆	⋆	⋆	⋆		⋆	6
Alonso de Lecinana et al. (40)		⋆	⋆	⋆	⋆	⋆	⋆	⋆	7
Moncayo et al. (22)	⋆	⋆	⋆	⋆		⋆		⋆	6
Schaller et al. (35)		⋆	⋆	⋆	⋆	⋆	⋆	⋆	7
Sitzer et al. (38)	⋆	⋆	⋆	⋆		⋆		⋆	6
Weber et al. (34)	⋆	⋆	⋆	⋆		⋆		⋆	6
Wegener et al. (23)		⋆	⋆	⋆	⋆	⋆		⋆	6
Weih et al. (21)		⋆	⋆	⋆	⋆	⋆		⋆	6
Zsuga et al. (36)	⋆	⋆	⋆	⋆		⋆	⋆	⋆	7

### Stroke Severity

**Figure 2:**
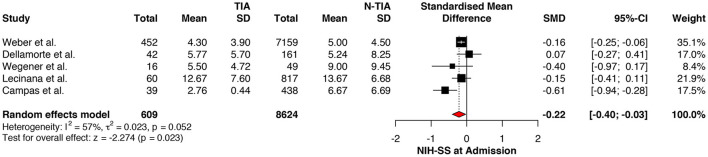
Comparison between TIA/non-TIA groups, NIHSS at admission.

**Figure 3:**
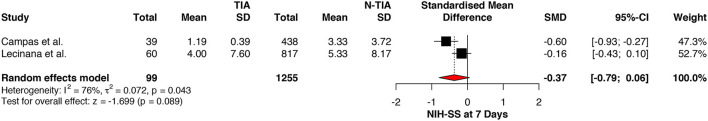
Comparison between TIA/non-TIA groups, NIHSS at 7 days.

The NIHSS scores of patients with prior TIAs were significantly lower at admission (MD: −0.22; 95%CI: −0.40 to −0.03, *P* = 0.023) (Figure 2), which faded away on re-assessment at 7 days (MD: −0.37; 95%CI: −0.79 to 0.06, *P* = 0.089) (Figure 3). However, significant heterogeneity was found among the studies reporting the NIHSS scores at 7 days (*I*^2^: 73%; *p* = 0.043) (Table 4).

**Table 4:** Summary of the patients' outcomes.

**Outcome**	**No of**	**Total, *N***	**TIA/Non-TIA**	**Random effect model**		
	**Studies no**.			**MD (95 % CI)**	**Test for overall effect**	**Heterogeneity**
						***I*^**2**^; *P*-value**
**NIH-SS score**						
At admission	5	9233	609/8624	−0.22 (−0.40; −0.03)	*P* = 0.023^*^	*I*^2^ = 57%; *P* = 0.052
At 7 days	2	1354	99/1255	−0.37 (−0.79; 0.06)	*P* = 0.089	*I*^2^ = 73%; *P* = 0.043^*^
**Length of stay, days**	3	6698	590/6108	−0.62 (−4.09; 2.85)	*P* = 0.725	*I*^2^ = 81%; *P* = 0.005^*^
**Glycemia**	3	3361	293/3068	−0.60 (−1.25; 0.06)	*P* = 0.074	*I*^2^ = 95%; *P* < 0.001^*^
**SBP**	2	2484	233/2251	0.02(−0.15; 0.20)	*P* = 0.788	*I*^2^ = 19%; *P* = 0.266
**DBP**	2	2484	233/2251	−0.07 (−0.29; 0.14)	*P* = 0.504	*I*^2^ = 39%; *P* = 0.201
**Outcome**	**No of**	**Total**, ***N***	**TIA/Non-TIA**	**Random effect model**		
	**Studies no**.			**OR (95% CI)**	**Test for overall effect**	**Heterogeneity**
						***I***^**2**^**;** ***P*****-value**
**Mortality**	5	5,109	524/4,585	0.88 (0.57; 1.34)	*P* = 0.545	*I*^2^ = 39%; *P* = 0.161
**Improvement**	2	1,025	97/928	1.43 (0.93; 2.21)	*P* = 0.106	*I*^2^ = 00%; *P* = 0.692
**TOAST**						
Large-artery atherosclerosis	7	16,419	1,224/15,195	1.67 (1.47; 1.90)	*P* < 0.001^*^	*I*^2^ = 00%; *P* = 0.548
Cardioembolism	7	16,419	1,224/15,195	0.65 (0.55; 0.76)	*P* < 0.001^*^	*I*^2^ = 09%; *P* = 0.358
Small-artery occlusion (lacune)	4	14,935	1,114/13,821	0.91 [0.69; 1.21]	*P* = 0.511	*I*^2^ = 62%; *P* = 0.048^*^
Other causes	7	16,419	1,224/15,195	0.87 (0.76; 1.00)	*P* = 0.043^*^	*I*^2^ = 00%; *P* = 0.942
**mRS at admission**						
mRS ≤3	2	12,408	784/11,624	1.13 (0.64; 1.99)	*P* = 0.675	*I*^2^ = 93%; *P* < 0.001^*^
mRS ≤1	2	12,408	784/11,624	1.15 (0.63; 2.12)	*P* = 0.647	*I*^2^ = 93%; *P* < 0.001^*^
**mRS at discharge**						
mRS ≤3	5	14,399	1,043/13,356	1.37 (1.14; 1.64)	*P* < 0.001^*^	*I*^2^ = 25%; *P* = 0.252
mRS 0–2	5	14,399	1,043/13,356	1.45 (1.16; 1.81)	*P* = 0.001^*^	*I*^2^ = 48%; *P* = 0.103
mRS ≤1	4	12,646	822/11,824	1.52 (1.11; 2.07)	*P* = 0.009^*^	*I*^2^ = 61%; *P* = 0.055^*^
**mRS at follow-up**						
mRS ≤3	2	1,484	110/1,374	1.64 (1.04; 2.59)	*P* = 0.034^*^	*I*^2^ = 02%; *P* = 0.362
mRS 0–2	2	1,007	71/936	1.40 (0.85; 2.31)	*P* = 0.181	*I*^2^ = 00%; *P* = 0.776

*Number of variation in NIHSS >8. DBP, diastolic blood pressure; SBP, systolic blood pressure; NIHSS, The National Institutes of Health Stroke Scale; mRS, The Modified Rankin Scale; MD, mean difference; OR, odds ratio*.

* *Statistically significant*.

### Clinical Outcomes

There was no significant difference between stroke patients with prior TIAs and stroke patients without prior TIA in terms of mortality (OR: 0.88; 95%CI: 0.57 to 1.34, *P* = 0.545) (Supplementary Figure 1). Besides, there were no statistically differences in terms of significant clinical improvement (variation in NIHSS >8) (OR: 1.43; 95%CI: 0.93 to 2.21, *P* = 0.106) (Supplementary Figure 2), length of stay (MD: −0.62; 95%CI: −4.09 to 2.85, *P* = 0.725) (Supplementary Figure 3), glycemia (MD: −0.60; 95%CI: −1.25 to 0.06, *P* = 0.074) (Supplementary Figure 4), systolic blood pressure (MD: −0.02; 95%CI: −0.15 to 0.20, *P* = 0.788) (Supplementary Figure 5), or diastolic blood pressure (MD: −0.07; 95%CI: −0.29 to 0.14, *P* = 0.504) (Supplementary Figure 6). Significant heterogeneity was recognized for studies that reported the length of stay and glycemia only (*I*^2^: 81%; *p* = 0.005, and *I*^2^: 95%; *p* < 0.001, respectively) (Table 4).

### TOAST Classification

We found that cardioembolism was significantly less prevalent in patients with TIAs while large vessel disease was significantly associated with increased incidence of cardioembolism (OR: 0.65; 95%CI: 0.55 to 0.76, *P* < 0.001) (Supplementary Figure 7) and (OR: 1.67; 95%CI: 1.47 to 1.90, *P* < 0.001) (Supplementary Figure 8), respectively. On the other hand, no statistical significance was found between the two groups in terms of lacunar stroke (OR: 1.23; 95%CI: 0.66 to 2.32, *P* = 0.516) (Supplementary Figure 9) or small vessel disease (OR: 0.91; 95%CI: 0.69 to 1.21, *P* = 0.511) (Supplementary Figure 10). No significant heterogeneity was found among all analyses, except for studies that reported small vessel disease only (*I*^2^: 62%; *p* = 0.042) (Table 4).

### Functional Outcomes

**Figure 4:**
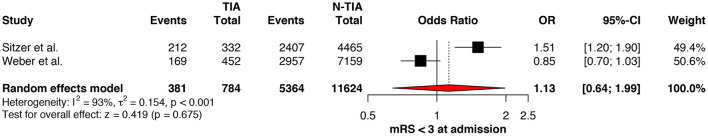
Comparison between TIA/non-TIA groups, mRS ≤3 at admission.

**Figure 5:**
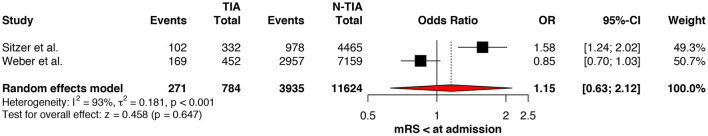
Comparison between TIA/non-TIA groups, mRS ≤1 at admission.

**Figure 6:**
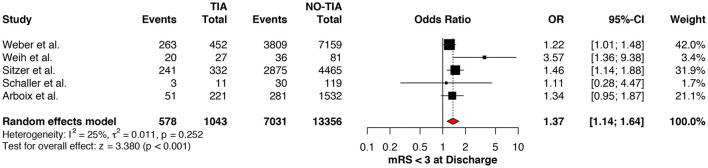
Comparison between TIA/non-TIA groups, mRS ≤3 at discharge.

**Figure 7:**
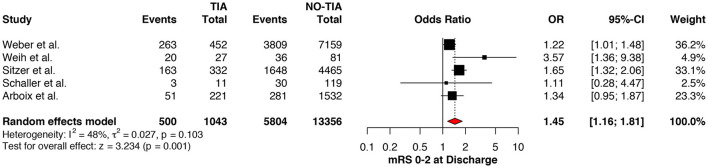
Comparison between TIA/non-TIA groups, mRS ≤2 at discharge.

**Figure 8:**
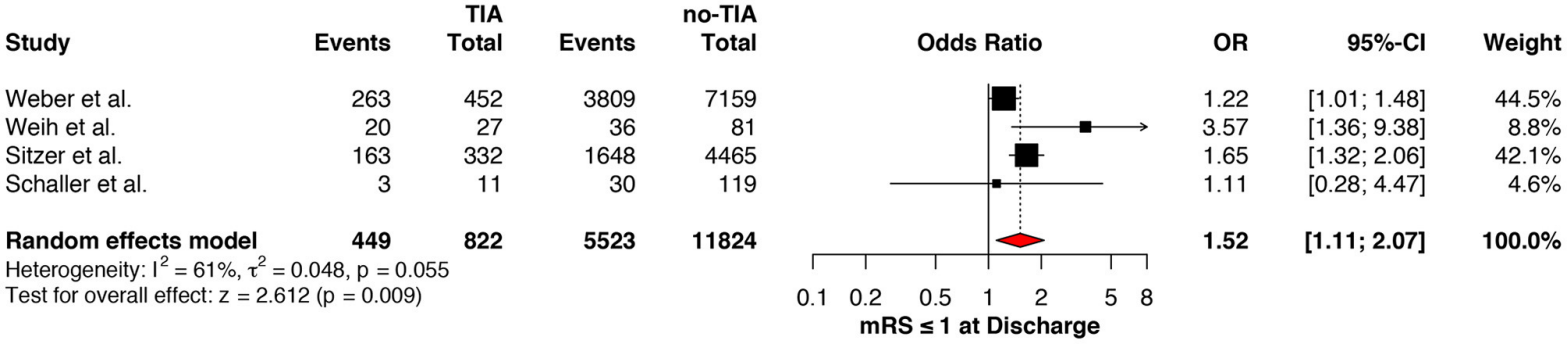
Comparison between TIA/non-TIA groups, mRS ≤1 at discharge.

**Figure 9:**
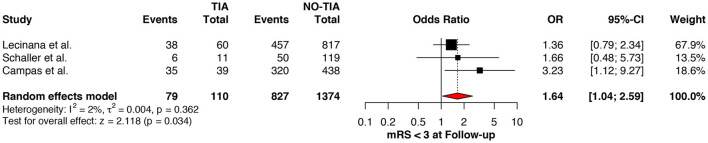
Comparison between TIA/non-TIA groups, mRS ≤3 at follow-up.

**Figure 10:**
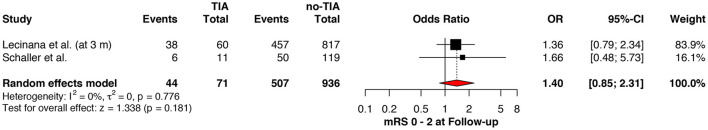
Comparison between TIA/non-TIA groups, mRS ≤2 at follow-up.

Regarding the mRS score at admission, there was no significant difference between the two groups, neither for mRS ≤3 (OR: 1.13; 95%CI: 0.64 to 1.99, *P* = 0.675) (Figure 4) nor for mRS ≤1 (OR: 1.15; 95%CI: 0.63 to 2.12, *P* = 0.647) (Figure 5). Significant heterogeneity was detected between the two studies which reported this outcome (*I*^2^: 93%; *p* < 0.001). On the other hand, the estimated mRS score at discharge was significantly lower in patients with TIAs, whether for mRS ≤3 (OR: 1.37; 95%CI: 1.14 to 1.64, *P* < 0.001) (Figure 6), mRS 0 to 2 (OR: 1.45; 95%CI: 1.16 to 1.81, *P* = 0.001) (Figure 7), or mRS ≤1 (OR: 1.52; 95%CI: 1.11 to 2.07, *P* = 0.009) (Figure 8), with estimated heterogeneity for studies that reported the latter outcome (*I*^2^: 61%; *p* = 0.055). At the last follow up report, compared with those who did not experience a prior TIAs, patients with prior TIAs showed higher rates of mRS ≤3 (OR: 1.64; 95%CI: 1.04 to 2.59, *P* = 0.034) (Figure 9). However, rates of mRS 0 to 2 (OR: 1.40; 95%CI: 0.85 to 2.31, *P* = 0.181) (Figure 10) were comparable among the two groups (Table 4).

## Discussion

Our results revealed that patients with TIAs prior to stroke events tend to have lower NIHSS scores on admission than those without prior TIAs. However, the NIHSS score was not significantly different between the two groups at 7 days. Furthermore, there was no statistically significant difference between the two groups in terms of mortality. It was also noted that despite the absence of differences in the admission mRS score between the two groups, patients with prior TIAs had lower mRS scores at discharge.

Stroke is a medical emergency associated with high rates of mortality and chronic disability (43). Therefore, it is crucial to identify the factors associated with better clinical outcomes after a stroke event, including pre and post-stroke factors. A TIA event is defined as a transient episode of focal neurologic deficits that happens due to focal ischemia affecting the brain, the spinal cord, or the retina without permanent tissue damage from the episodic ischemia (44). Transient ischemic attack is associated with an increased risk of ischemic stroke in the first 24–48 h after the TIA or during the following few months (44, 45). However, there has been a growing body of evidence in the medical literature that TIAs occurring before ischemic strokes might provide some neuroprotection, leading to better clinical outcomes (22, 46). The general principle that is implicated in this kind of neuroprotection is called IPC, which is defined as transient episodes of ischemia that alleviate the damage caused by a subsequent longer ischemic episode (47).

The concept of IPC or ischemic tolerance has been researched widely, and there are multiple mechanisms suggested being involved in the physiology of this phenomenon. These mechanisms act on both vascular and cellular levels. The recruitment of the collateral vascular pathways resulting from the ischemic events in TIA patients is hypothesized to produce neuroprotection that reduces the neuronal damage taking place when ischemic insults of longer durations occur in those patients (22, 33). Furthermore, TIAs might lead to decreased metabolic rates in the affected brain tissue without impaired coupling between the cerebral blood flow (CBF) and the metabolic rate. Thus, the brain areas affected by the TIA would be more conditioned to decreased CBF via decreased metabolic rate, which makes these areas eventually less prone to ischemic damage when exposed to longer-duration ischemia (35, 48). There is evidence that some TIA patients might have silent ischemic injuries, as shown by imaging studies, but without permanent neurological deficits (49). Thus, TIAs can be associated with subtoxic ischemic damage that is not clinically symptomatic but can activate a cascade of endogenous neuroprotective changes. These changes can improve the neurologic outcomes from subsequent stroke events (36).

On cellular and molecular levels, there are a few mechanisms that might contribute to the IPC in patients with TIAs prior to the stroke events compared with those without TIA history. For example, a few comparative studies demonstrated that patients with TIAs prior to strokes have lower levels of specific inflammatory markers that are known to get elevated as a part of the brain tissue response to ischemia in comparison with patients without TIAs (37, 41). Accordingly, it is suggested that TIAs boost the brain capacity to downregulate inflammatory responses, limiting the inflammatory response triggered by ischemic strokes. Consequently, this can lead to better clinical outcomes (41). Additionally, there are a few other mechanisms of ischemic tolerance that act on cellular and subcellular levels, including new protein synthesis, activation of adenosine A1 receptors leading to the opening of adenosine-triphosphate-dependent potassium channels, stimulation of NMDA receptors, upregulation of antioxidant enzymes, and overexpression of the immediate early genes and apoptosis suppression genes (22, 33, 50). Also, it has to be taken into consideration that TIAs can influence the clinical outcome of subsequent ischemic strokes by limiting the extent of reperfusion injury in patients receiving thrombolysis treatment (23, 35, 40).

While these several mechanisms are thought to be implicated in the IPC induced by TIA, there are a few points to be considered before drawing a conclusion in this regard. Firstly, a significant proportion of the TIA patients are started on secondary prevention treatments, including antiplatelet medications and anticoagulants. However, Deplanque et al. (51) found that prior TIA is associated with less severity of subsequent cerebral ischemia independently from the secondary prevention measures. Secondly, TIA patients might be more aware of their symptoms; they may seek medical advice and be hospitalized when they develop the subsequent acute ischemic stroke (AIS), which implies faster access to treatment than stroke patients without prior history of TIA (40). Moreover, the different mechanisms of stroke in TIA patients might act as a confounding factor since TIA was more common in patients who have intracranial atherosclerosis when compared with patients with cardioembolism (37, 41). Knowing that cardioembolism is associated with larger-size ischemic brain infarcts as compared with atherosclerosis-related strokes (52, 53), it could be assumed that patients with stroke due to atherosclerotic etiology might have a better functional outcome than patients with stroke from a cardioembolic source due to the larger size of the cardioembolic strokes, and the higher availability of collateral vascular pathways in patients with atherosclerosis (53) regardless of the neuroprotective of TIA. In this regard, a few studies accounted for these differences between strokes due to atherosclerotic disease and cardioembolic ones, where TIA was still associated with a better functional outcome in both types of stroke (33, 45).

Although several observational studies showed a positive impact of a prior TIA on the neurologic functional outcome of a following ischemic event (21, 22, 33–38, 41, 45, 51, 54, 55), there are a few reports that failed to confirm this (39, 40). Our pooled analysis showed a statistically significant difference between TIA patients and non-TIA patients regarding the initial NIHSS score after a stroke event and the mRS scores on discharge. Yet, there was no significant difference between the two groups in the NIHSS score at 7 days from the stroke event, admission mRS score, and mortality. Several factors would explain why TIA might not have a neuroprotective effect against subsequent ischemic stroke in certain patients, which was reflected in the abovementioned studies. They would also explain why our meta-analysis found significant differences in NIHSS score only at admission after stroke events but not at 7 days from the stroke event. Age is a very crucial factor that might contribute to the less neuroprotective effect of TIA on subsequent stroke. Della Morte et al. (39) found that TIA is not associated with any significant difference in NIHSS and mRS scores, whether on admission or discharge, between TIA (within 72 h prior to stroke) and non-TIA patients at the age of ≥65 years. This might be due to the aging-related downregulation of the mechanisms involved in IPC, including mitochondrial functioning, which might lead to an increased burden of oxidative stress and increased toxicity from excessive intracellular calcium influx (56, 57). Huang et al. (55) compared the TIA with the non-TIA patients in terms of neurological deficiency after a stroke event, and the subgroup analysis indicated that there is a neuroprotective effect of TIA that significantly improves the clinical outcome of ischemic stroke, but this effect disappears in patients >75 years old. It might also be that the tissue degeneration associated with aging can lead to decreased tissue response to ischemia leading to diminished ischemic tolerance in elderly patients (55). An animal study by Kato et al. (16) demonstrated that the ischemic tolerance induced by transient ischemia takes around 24 h to develop and its impact lasts for about 1 week. In the observational study conducted by Moncayo et al. (22), it was revealed that the interval between the TIA and stroke events factors in the extent of the TIA's neuroprotective effect as favorable outcomes were associated with less time elapsed between the TIA and the subsequent stroke event. A similar association was noticed in the observation study conducted by Castillo et al. (37). Thus, the interval variability between the two events among the different studies could partially account for the differences noticed in these studies' results. The TIA duration could also influence the TIA-induced in addition to the interval between TIA and subsequent stroke. It was reported that TIAs lasting more than 20 min were not associated with a better outcome than that seen in non-TIA patients (22). The side of the TIA and subsequent AIS event, being ipsilateral vs. contralateral, might be another factor to consider while studying the TIA-induced IPC. It is imperative to research further the extent of the ischemic tolerance induced by TIA to understand whether a TIA on one side triggers IPC globally or only on the side where it takes place. According to stroke events being ipsilateral or contralateral to the preceding TIA, there could be some differences in the inflammatory response induced by these ischemic events (37). Not all the studies included in our meta-analysis took that factor into consideration which might cloud the actual impact of the TIA on neurologic functioning after a stroke. The ischemic tolerance mediated by TIA might vary according to ischemic stroke being superficial or subcortical. Transient ischemic attack showed a neuroprotective effect on superficial infarcts. Still, this effect was absent in patients with subcortical stroke, which could stem from the fact that subcortical strokes are already less commonly associated with the poor functional outcome, which can lead to less evidently seen neuroprotective effect in TIA patients with subcortical strokes as compared with those with superficial strokes (22). The accuracy of TIA diagnosis is another critical point that can affect the quality of the retrospective studies looking at the neuroprotective effect of TIA.

## Study Limitation

We were unable to do subgroup analyses due to the lack of subgroup data in the included studies, which is considered a limitation, given the abovementioned factors that might have impacted the effect of TIAs on subsequent strokes. Moreover, the nature of the included studies may affect the quality of the evidence, considering that most of the studies have a retrospective design, and all included studies were conducted in European countries.

## Conclusion

Understanding the physiological basis of IPC that TIAs might mediate can open the door for new preventive treatment modalities for patients at high risk of developing ischemic strokes. A few randomized clinical trials (RCTs) have tested the efficacy of remote ischemic conditioning (RIC) in preventing and/or treating ischemic stroke. Remote ischemic conditioning in these studies was generally done by limiting the blood follow extremities, then releasing the blood from areas exposed to ischemia in an attempt to induce a neuroprotective effect centrally (58–64). A meta-analysis of these RCTs showed that RIC might have a beneficial effect in preventing and treating ischemic stroke (65). However, the evidence provided in this meta-analysis was of low quality, and further research is needed in this area to confirm the efficacy of RIC in preventing and treating ischemic stroke.

## Data Availability Statement

The original contributions presented in the study are included in the article/Supplementary Material, further inquiries can be directed to the corresponding author/s.

## Author Contributions

DK, DH, and SG were responsible for the idea and the study design. All authors extracted the data. SK and SG performed the analysis. All authors shared in the manuscript writing, formatting, and approval of the final version.

## Conflict of Interest

KK was employed by companies Nested Knowledge and Superior Medical Experts. The remaining authors declare that the research was conducted in the absence of any commercial or financial relationships that could be construed as a potential conflict of interest.

## Publisher's Note

All claims expressed in this article are solely those of the authors and do not necessarily represent those of their affiliated organizations, or those of the publisher, the editors and the reviewers. Any product that may be evaluated in this article, or claim that may be made by its manufacturer, is not guaranteed or endorsed by the publisher.

## Abbreviations

CBF: cerebral blood flow
IPC: ischemic preconditioning
MD: mean difference
CI: confidence interval
mRS: modified Rankin scale
NIHSS: National Institute of Health Stroke Scale
OR: odds ratio
RCT: randomized clinical trial
RIC: remote ischemic conditioning
TIA: Transient ischemic attack
AIS: acute ischemic stroke.
