# Supplementary figures and images for: Transient Ischemic Attacks Preceding Ischemic Stroke and the Possible Preconditioning of the Human Brain: A Systematic Review and Meta-Analysis
Source: Front Neurol. 2021 Nov 24;12:755167. doi: 10.3389/fneur.2021.755167 (PMC8652229; doi:10.3389/fneur.2021.755167)

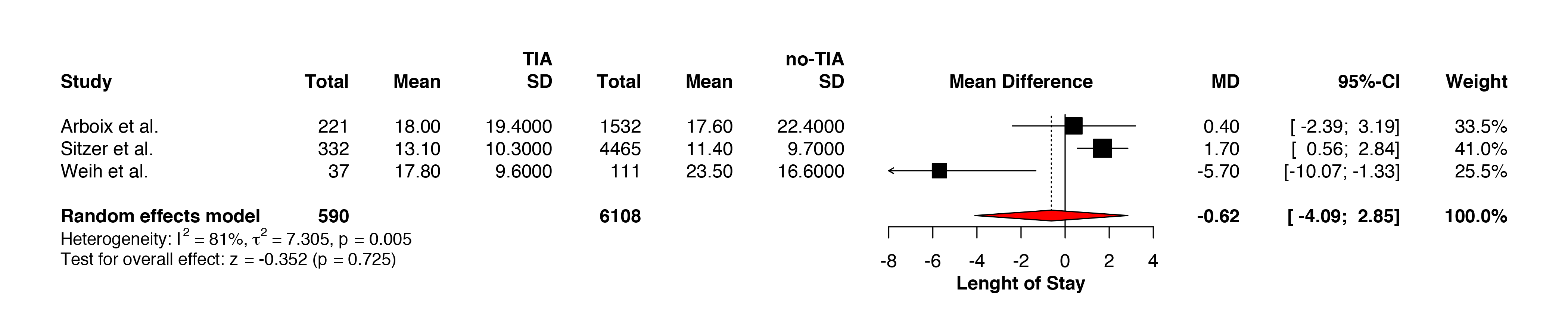

Supplement: Supplementary file 1 [file Image_1.TIF]

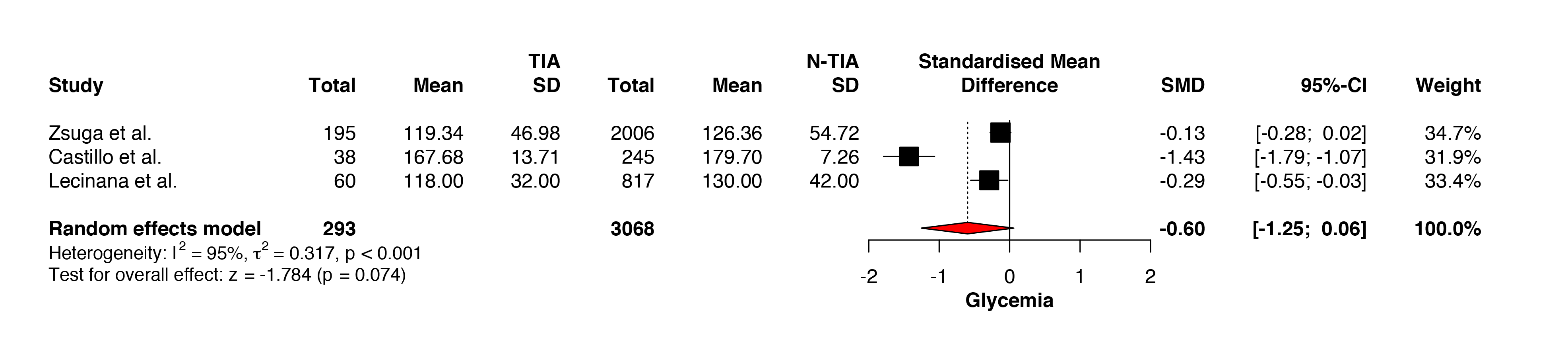

Supplement: Supplementary file 2 [file Image_2.TIF]

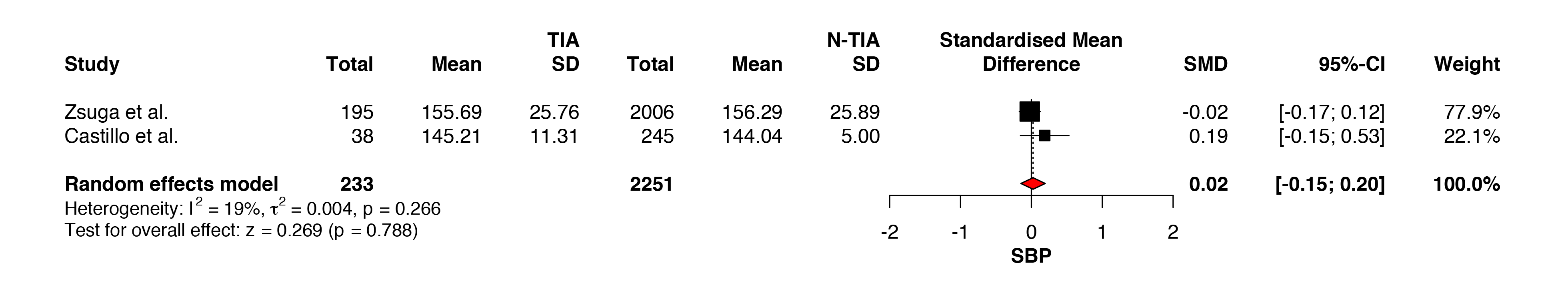

Supplement: Supplementary file 3 [file Image_3.TIF]

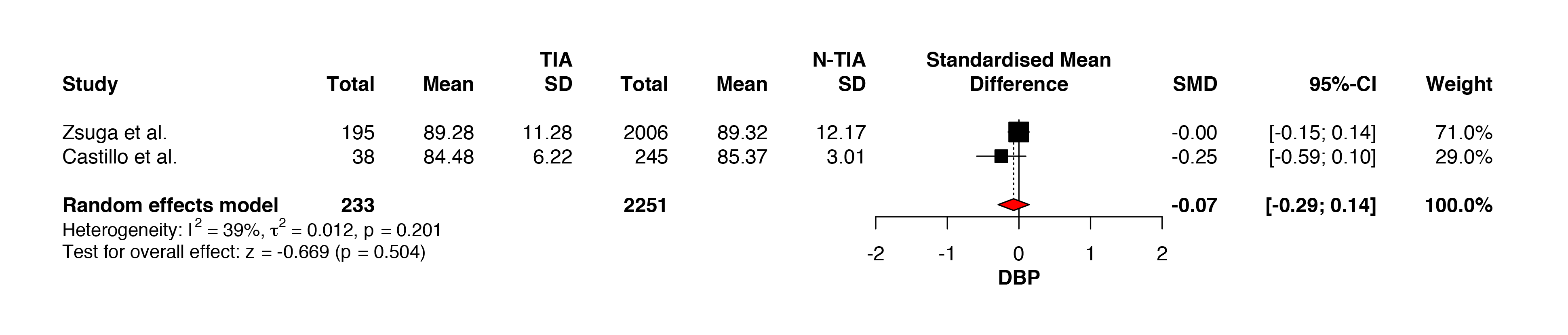

Supplement: Supplementary file 4 [file Image_4.TIF]

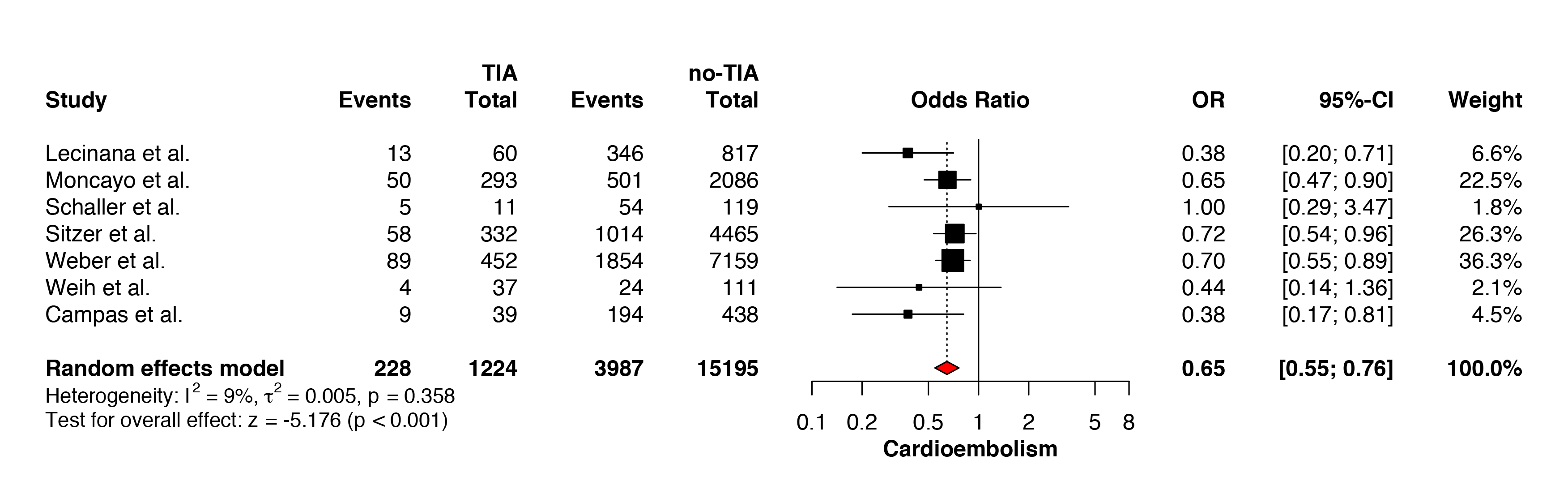

Supplement: Supplementary file 5 [file Image_5.TIF]

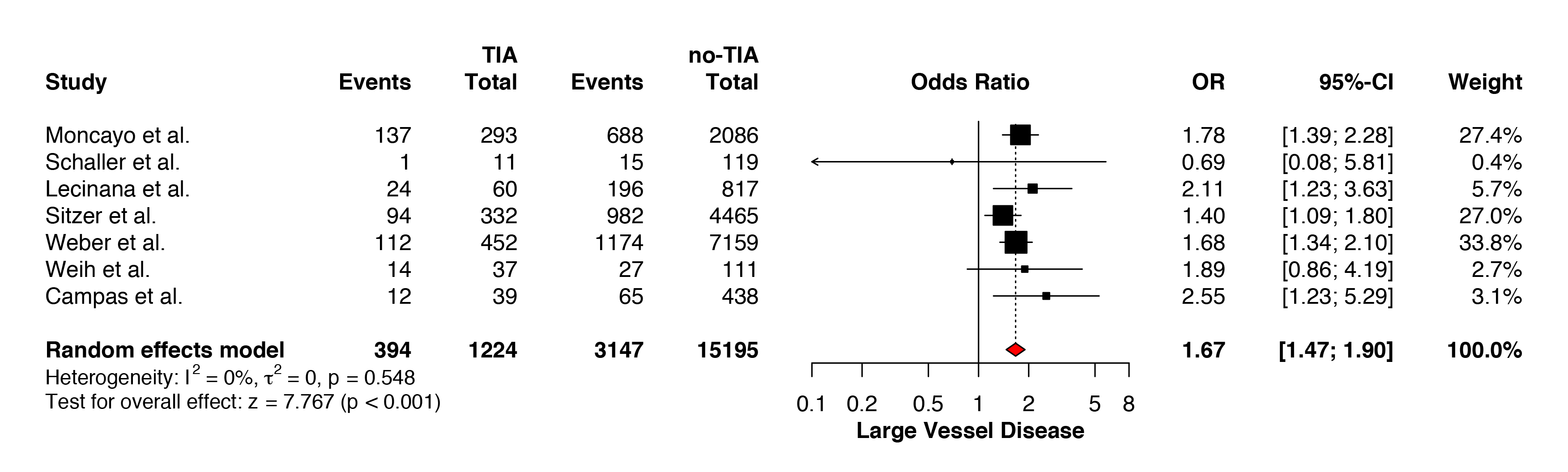

Supplement: Supplementary file 6 [file Image_6.TIF]

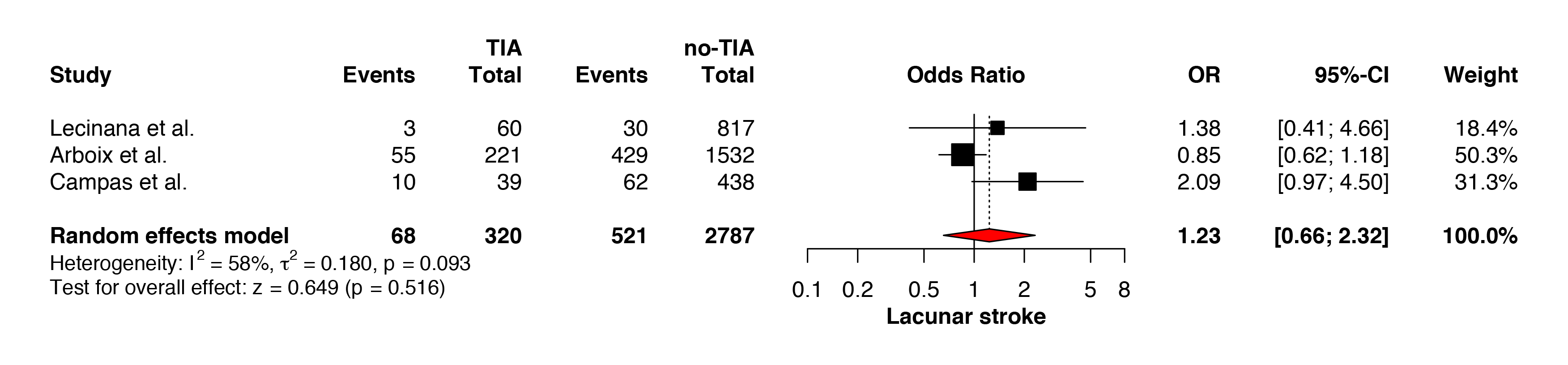

Supplement: Supplementary file 7 [file Image_7.TIF]

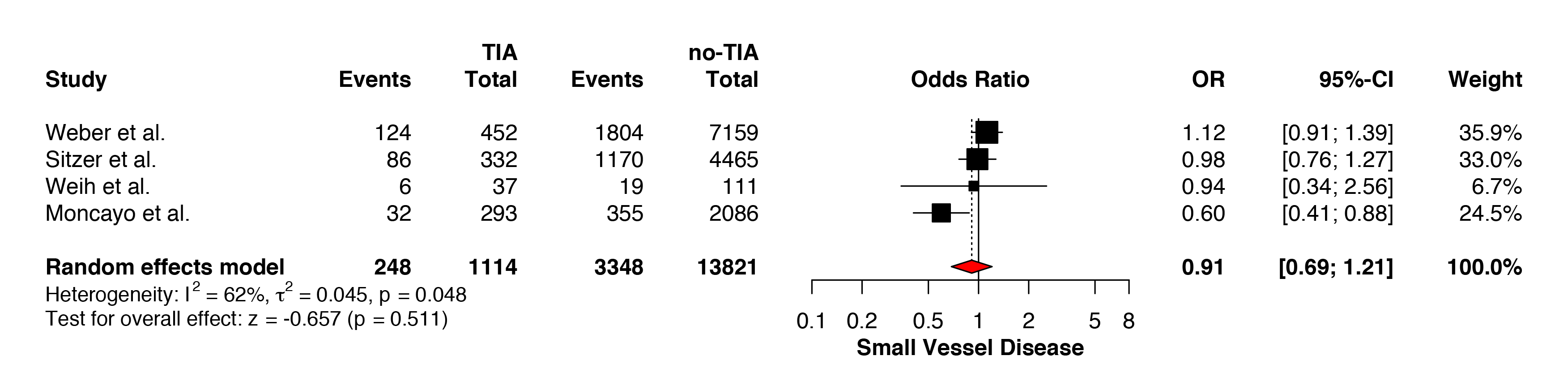

Supplement: Supplementary file 8 [file Image_8.TIF]

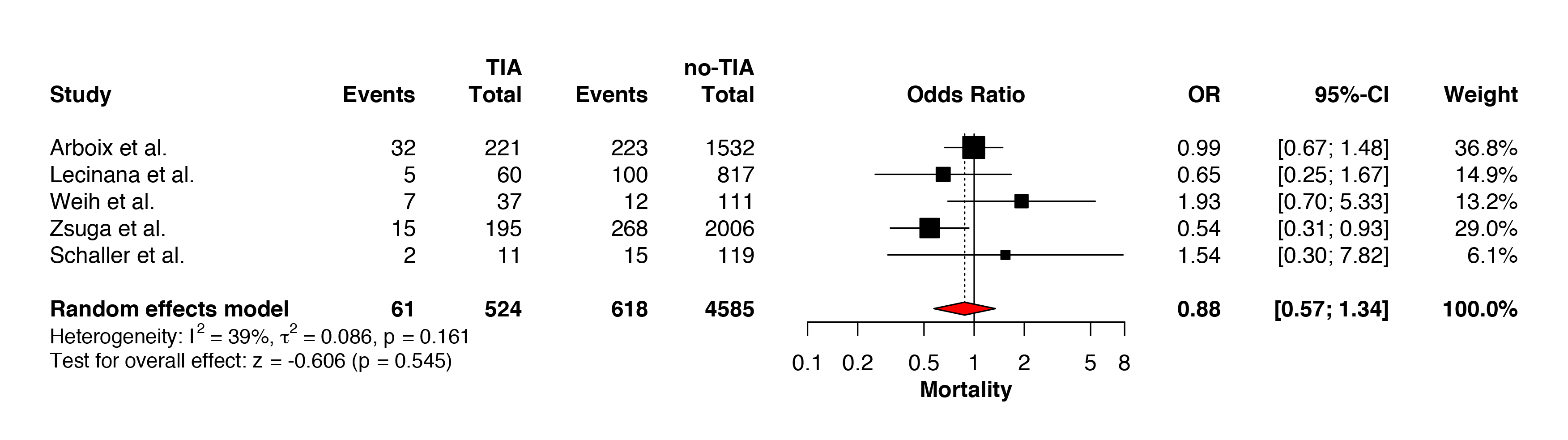

Supplement: Supplementary file 9 [file Image_9.TIF]

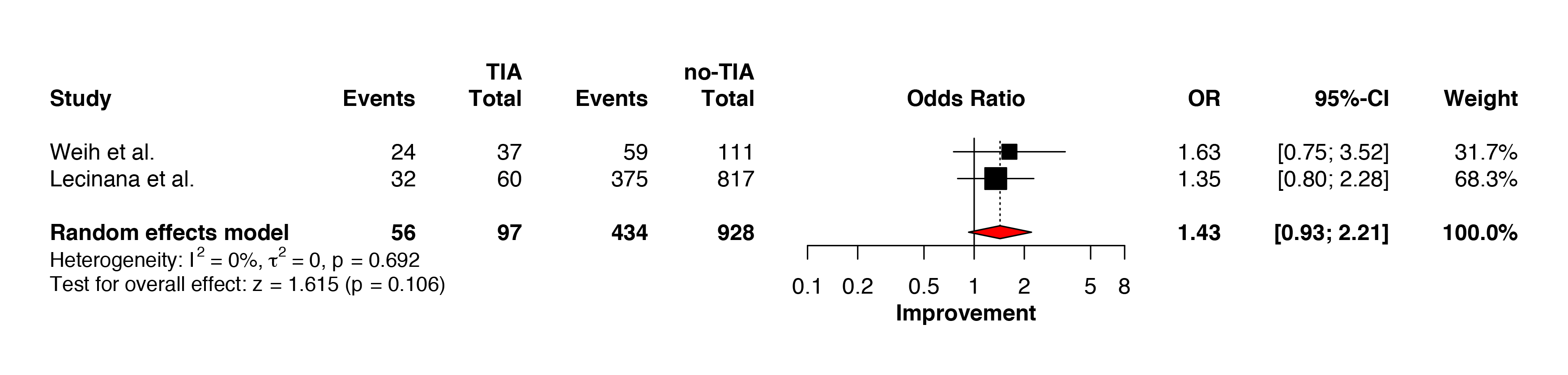

Supplement: Supplementary file 10 [file Image_10.TIF]
